# The role of causal criteria in causal inferences: Bradford Hill's "aspects of association"

**DOI:** 10.1186/1742-5573-6-2

**Published:** 2009-06-17

**Authors:** Andrew C Ward

**Affiliations:** 1 Minnesota Population Center, 50 Willey Hall, 225 – 19th Avenue South, University of Minnesota, Minneapolis, MN 55455, USA

## Abstract

As noted by Wesley Salmon and many others, causal concepts are ubiquitous in every branch of theoretical science, in the practical disciplines and in everyday life. In the theoretical and practical sciences especially, people often base claims about causal relations on applications of statistical methods to data. However, the source and type of data place important constraints on the choice of statistical methods as well as on the warrant attributed to the causal claims based on the use of such methods. For example, much of the data used by people interested in making causal claims come from non-experimental, observational studies in which random allocations to treatment and control groups are not present. Thus, one of the most important problems in the social and health sciences concerns making justified causal inferences using non-experimental, observational data. In this paper, I examine one method of justifying such inferences that is especially widespread in epidemiology and the health sciences generally – the use of causal criteria. I argue that while the use of causal criteria is not appropriate for either deductive or inductive inferences, they do have an important role to play in inferences to the best explanation. As such, causal criteria, exemplified by what Bradford Hill referred to as "aspects of [statistical] associations", have an indispensible part to play in the goal of making justified causal claims.

## Introduction

As noted by Salmon [1] and others [2,3], causal concepts are ubiquitous in every branch of theoretical science, in the practical disciplines and in everyday life. In the case of the social sciences, Marini and Singer write that "the identification of genuine causes is accorded a high priority because it is viewed as the basis for understanding social phenomena and building an explanatory science" [4]. Although health services research is not so interested in "building an explanatory science", it too, like the social sciences with which it often overlaps, sets a premium on identifying genuine causes [5]. Establishing "an argument of causation is an important research activity," write van Reekum et al., "because it influences the delivery of good medical care" [6]. Moreover, given the keen public and political attention given recently to issues of health care insurance and health care delivery, a "key question" for federal, state and local policy makers that falls squarely within the province of health services research is how much an effect different kinds of health insurance interventions have on people's health, "and at what cost" [7].

This focus on causality and causal concepts is also pervasive in epidemiology [8-14], with Morabia suggesting that a name "more closely reflecting" the subject matter of epidemiology is "'population health etiology', etiology meaning 'science of causation"' [15]. For example, Swaen and Amelsvoort write that one "of the main objectives of epidemiological research is to identify causes of diseases" [16], while Botti, et al. write that a "central issue in environmental epidemiology is the evaluation of the causal nature of reported associations between exposure to defined environmental agents and the occurrence of disease. [17]" Gori writes that epidemiologists "have long pressed the claim that their study belongs to the natural sciences ... [and seek] to develop theoretical models and to identify experimentally the causal relationships that may confirm, extend, or negate such models" [18], and Oswald even goes so far as to claim that epidemiologists are "obsessed with cause and effect. [19]" Of course, it is true that some writers [20] are a bit more cautious when describing how considerations of causality fit into the goals of epidemiology. Weed writes that the "purpose of epidemiology is *not *to prove cause-effect relationships ... [but rather] to acquire knowledge about the determinants and distributions of disease and to apply that knowledge to improve public health. [21]" Even here, though, what seems implicit is that establishing cause-and-effect relationships is still the ideal goal of epidemiology, and as Weed himself writes in a later publication, finding "a cause, removing it, and reducing the incidence and mortality of subsequent disease in populations are hallmarks of public health and practice" [22].

Often people base claims about the existence and strength of causal relations on applications of statistical methods to data. However, the source and type of data place important constraints on the choice of statistical methods as well as on the warrant attributed to the causal claims based on the use of such methods [23]. In this context, Urbach writes that an "ever-present danger in ... investigations is attributing the outcome of an experiment to the treatment one is interested in when, in reality, it was caused by some extraneous variation in the experimental conditions" [24]. Expressed in a counterfactual framework, the danger is that while the causal contrast we want to measure is that between a target population under one exposure and, counterfactually, that same population under a different exposure, the observable substitute we use for the target population under the counterfactual condition may be an imperfect substitute [25,26]. When the observable substitute is an imperfect substitute for the target population under the counterfactual condition, the result is confounding, and the measure of the causal contrast is confounded. In order to address this "ever-present danger", many users of statistical methods, especially those of the Neyman-Pearson or Fisher type [27,28], claim that randomization is necessary.

Ideally, what randomization (random allocation to treatment and control or comparison groups) does is two-fold. First, following Greenland, the average of many hypothetical repetitions of a randomized control trial (RCT) will make "our estimate of the true risk difference statistically unbiased, in that the statistical expectation (average) of the estimate over the possible results equals the true value" [29]. In other words, randomization addresses the problem of statistical bias. However, as pointed out by Greenland [29], without some additional qualification, an ideally performed RCT does not "prevent the epidemiologic bias known as confounding" [29]. To reduce the probability of confounding, idealized random allocation must be used to create sufficiently large comparison groups. As Greenland notes, by using "randomization, one can make the probability of severe confounding as small as one likes by increasing the size of the treatment cohorts" [29]. For example, using the example in Greenland, Robins and Pearl, suppose that "our objective is to determine the effect of applying a treatment or exposure x_1 _on a parameter μ of the distribution of the outcome y in population A, relative to applying treatment or exposure x_0_" [30]. Further, let us suppose that "μ will equal μ_A1 _if x_1 _is applied to population A, and will equal μ_A0 _if x_0 _is applied to that population" [30]. In this case, we can measure the causal effect of x_1 _relative to x_0 _by μ_A1_-μ_A0_. However, we cannot apply both x_1 _and x_0 _to the same population. Thus, if A is the target population, what we need is some population B for which μ_B1 _is known to equal (has a high likelihood of equaling) μ_A1_, and some population C for which μ_C0 _is known to equal (has a high likelihood of equaling) μ_A0_. To create these two groups, we randomly sample from A. If the randomization is ideal **and **the treatment cohorts (B and C) are sufficiently large, then we can expect, in probability, that the outcome in B would be the outcome if everyone in A were exposed to x_1_, while the outcome in C would be the outcome if everyone in A were exposed to x_0_. Thus, what idealized randomization does, when the treatment cohorts created by random selection from the target population are sufficiently large, is to create two sample populations that are exchangeable with A under their respective treatments (x_1 _and x_0_). In this way, a sufficiently large, perfectly conducted RCT controls for confounding, in probability, because the randomized allocation into B and C is, in effect, random sampling from the target population A to create reference populations B and C that are exchangeable with A. As Hernán notes, in "ideal randomized experiments, association *is *causation" [31].

Hernán's claim that in idealized randomized experiments, "association *is *causation", is a contemporary restatement of a view presented earlier by the English statistician and geneticist R. A. Fisher. According to Fisher, "to justify the conclusions of the theory of estimation, and the tests of significance as applied to counts or measures arising in the real world, it is logically necessary that they too must be the results of a random process" [32]. It is this contention, captured succinctly by Hernán, that is the centerpiece of the widely held belief that randomized clinical trials (RCTs) are, and ought to be, the "gold standard" of evaluating the causal efficacy of interventions (treatments) [33-36]. Thus, Machin writes that it is likely that "the single most important contribution to the science of comparative clinical trials was the recognition more than 50 years ago that patients should be allocated the options under consideration at random [37]. Similarly, while she believes that the value of RCTs depends crucially on the subject matter and the assumptions one is willing to make [38], Cartwright notes that many evidence-based policies call for scientific evidence of efficacy before being agreed to, and that government and other agencies typically claim that the best evidence for efficacy comes from RCTs [39].

Although generally considered the gold standard of research whose goal is to make justified causal inferences, it should come as no surprise that there is a variety of limitations associated with the use of RCTs. Some of these limitations are practical. For example, not only are RCTs typically expensive and time-consuming, there are important ethical questions raised when needed resources, that are otherwise limited or scarce, are randomly allocated. Similarly, it seems reasonable to worry about the ethical permissibility of an RCT when its use requires withholding a potentially beneficial treatment from people who might otherwise benefit from being recipients of the treatment. In addition to these practical concerns, there is also a variety of methodological limitations. Even if an idealized RCT is internally valid, generalizations from it to a wider population may be very limited. As noted by Silverman, a "review of epidemiological data and inclusion and exclusion criteria for trials of antipsychotic treatments revealed that only 632 of an estimated 36,000 individuals with schizophrenia would meet basic eligibility requirements for participation in a randomized controlled experiment" [40]. In such cases, even if there are no problems with differential attrition, the exportation of a finding from the experimental population to a target population may well go beyond what is justified by the use of RCTs. Even more generally, there is no guarantee either that the observable substitute for the target population under the counterfactual condition is a "good" substitute, or that a single RCT will result in a division in which possible confounders of the measured outcome are randomly distributed. Regarding the latter point, Worrall remarks that even for an impeccably designed and carried out RCT, "all agree that in any particular case this may produce a division which is, once we think about it, imbalanced with respect to some factor that plays a significant role in the outcome being measured" [41]. While it may be possible to reduce the probability of such baseline imbalances by multiple repetitions of the RCT, these repetitions, whose function is to give the limiting average effect [42], may not be practically feasible. Moreover, at least when the repetitions are "real life" repetitions and not computer simulations, there is no reason to believe that each of the repetitions will be "ideal", and more reasons to believe that they will not all be ideal. For this reason, multiple (real life) repetitions of the RCT are more likely to increase the likelihood of other kinds of bias, such as differential attrition, not controlled for by use of an RCT.

Of course there are a variety of approaches that one can take in attempting to meet these, and other limitations of RCTs. While not intending to downplay the importance of RCTs and the attempts to address the limitations associated with their use, much of the data used by people interested in making causal claims do not come from experiments that use random allocation to control and treatment or comparison groups. Indeed, as Herbert Smith writes, few "pieces of social research outside of social psychology are based on experiments" [43]. Thus, one of the most important problems in the social and health sciences, as well as in epidemiology, concerns whether it is possible to make warranted causal claims using non-experimental, observational data. The focus on observational data, as opposed to experimental data, leads us away from RCTs and towards an examination of what Weed has called the "most familiar component of the epidemiologist's approach to causal inference", viz., "causal criteria" [44]. In the context suggested by the quotation from Weed, the argument presented in this paper has three parts. First, I argue that, properly understood, causal inferences that make use of causal criteria, exemplified by the Bradford Hill "criteria", are neither deductive nor inductive in character. Instead, such inferences are best understood as instances of what philosophers call "inference to the best explanation". Second, I argue that even understood as components of an inference to the best explanation (the causal claim being the best explanation), causal criteria have many problems, and that the inferences their use sanctions are, at best, very weak. Finally, I conclude that while the inferential power of causal criteria is weak, they still have a pragmatic value; they are tools, in the toolkits of people interested in making causal claims, for preliminary assessments of statistical associations. To vary a remark by Mazlack about "association rules", while satisfactions of causal criteria (such as the Bradford Hill criteria with which this paper principally deals) do not warrant causal claims, their judicious application is important and, perhaps in many cases, indispensible for identifying interesting statistical relationships that can then be subjected to a further, more analytically rigorous statistical examination [45].

## Analysis

Relative to RCTs, the absence of random allocation to treatment and control or comparison groups is what leads to one of, though not all of, the most important methodological issues observational, non-experimental studies face. In the absence of randomized allocations from a sufficiently large population to treatment and control or comparison groups, we no longer have a probabilistic guarantee that there is no statistical bias and that we have minimized the probability of confounding. Thus, because there is no random allocation in an observational study, and because, as noted by Little and Rubin, without "a model for how treatments are assigned to units, formal causal inference, at least using probabilistic statements, is impossible" [46], some other method of allocation (and set of assumptions) is needed for observational studies. One possibility, according to Little and Rubin, is that researchers may statistically control for "recorded confounders" and then assume, either explicitly or implicitly, that the non-randomized "treatment assignment corresponds to that of an unconfounded randomized experiment" [46]. A problem with this method is that the assumption is not testable, and frequently made without any good theoretical support. Nevertheless, while observational studies may take a variety of different forms, they do all share an important characteristic with RCTs; viz., all those non-statistical aspects of RCTs, apart from their use of randomized allocation, that go towards making them well-designed experiments and contribute to causal inferences, are also important in well-designed observational, non-experimental studies from which causal inferences are drawn. Put a bit more precisely, any non-statistical characteristic whose presence is, in the case of RCTs, necessary for a well-founded causal inference to a causal claim (e.g. compliance to assigned treatments by subjects, any missing data having the same distribution as observed data) is also necessary for a well-founded causal inference in the case of observational, non-experimental studies. Thus, as William Cochran who, according to Rosenbaum, was one of the first to present observational studies "as a topic defined by principles and methods of statistics" [47] remarks, "to a large extent, workers in observational research have tried to copy devices that have proved effective in controlled experiments" [48].

However, suppose that one is not willing to assume that the non-randomized treatment cohort in an observational study "corresponds" to the treatment cohort in an unconfounded randomized experiment using the same sample (study) population. In this case, assurances that the non-statistical characteristics of a well-designed and executed RCT are also present in the observational study are not sufficient to make well-founded causal inferences from the observational data. Something more is needed. It is at this point that people interested in making well-founded causal inferences based on observational data differ in their methodological approaches. One approach is to use one or more appropriately chosen statistical methods to model observational data in such a way that the RCT interventionist method of random allocations into treatment and control or comparison groups is, in one way or another, captured by the characteristics of the model. This is the idea behind Rubin's claim that an "observational study should be conceptualized as a broken randomized experiment" that we use statistical methods to fix as best we can [49], and Freedman's similar remark that "one objective of statistical modeling is to create an analogy, perhaps forced, between an observational study and an experiment" [50]. For example, a method widely used in epidemiology, the social sciences and health services research to capture observed imbalances in covariate patterns among groups, and so justify inferences that changes in one or more independent variables cause changes in a dependent variable, is to use regression models [51,52]. According to Clogg and Haritou, one of the central underlying assumptions in what they refer to as the "regression method of causal inference" is that "experimental manipulation or control through randomization can be replaced by *statistical control *or partialing with a regression model, along with a few assumptions that seem benign to most researchers" [53]. Whether those "few assumptions" (e.g. assumptions about functional form, what variables to include or exclude from the regression equation [54] and random allocation of treatment within strata for the controlled variables) are genuinely plausible and "benign" in most real-world situations is a matter of some debate [51].

More recently, propensity score estimation (using regression as part of the process, but with no attempt to interpret regression coefficients causally) and matching has emerged as a method to warrant claims about average causal effects and average causal effects on the treated [49,55,56]. Introduced by Paul Rosenbaum and David Rubin in 1983, the propensity score is the conditional property of a subject/unit in a sample (study) population being exposed or treated, given a set of observed covariates that one believes predicts the exposure or treatment [57]. The idea, roughly, is that once we have the estimated propensity score, we can match "subjects in exposed and unexposed conditions on their propensity scores" [58]. On the assumption that the matched samples are balanced with respect to the set of observed covariates, and on the further assumption, questioned by some, that "if both subjects have the same [estimated] probability of exposure, it is random which one was in fact exposed and which was not", we have simulated random allocation [59]. After this, it is a relatively straightforward exercise to estimate a causal effect of exposure or treatment [59]. Like the use of regression models to estimate causal effects, the use of propensity scores and matching (or some other methods such as stratification or weighting on the propensity score) to estimate causal effects makes a number of assumptions. For example, as suggested above, using propensity scores to address problems of statistical bias and confounding requires assuming that population members with similar estimated probabilities of exposure are exchangeable with respect to disease (outcome) frequency. Depending on the observational study, these assumptions either may be implausible or may place serious limitations what causal inferences one can justifiably make [58,59].

There is, to be sure, much to value in approaching questions of causality in terms of fitting statistically well-defined models to the available data. In this connection, Heckman writes that a "major contribution of twentieth century econometrics was the recognition that causality and causal parameters are most fruitfully defined within formal economic models and that comparative statistics within these models ... most clearly define causal parameters [60]. Similarly, while acknowledging "statistical associations do not *logically *imply causation," Pearl claims that under "the assumptions of model minimality (and/or stability), there are patterns of dependencies that should be sufficient to uncover genuine causal relationships" [61]. However, at least in the case of observational studies, not everyone is sanguine about the use of statistically well-defined models to answer questions about the presence and relative strength of cause-and-effect relationships. Part of the reticence to embracing statistically based causal inferences is the worry that these kinds of inferences presuppose that the statistically modeled data are the products of randomized allocation, while part of the worry is that statistical modeling, by itself, cannot justify making causal inferences without the addition of non-statistically based assumptions. For example, Pearl writes that in those studies in which there is no random allocation (what he refers to as "imperfect experiments") "reasonable assumptions about the salient relationships in the domain" must be used to determine bounds of the causal effect of an exposure or treatment [61]. Freedman's criticism of Spirtes, Glymour and Scheines' attempt [62] to discover causal relationships by the use of directed graphs to represent statistical independence and dependence relationships between variables used in the graph makes an analogous point. According to Freedman, while the use of directed graphs and the associated algorithms by Sprites, Glymour and Scheines has "some technical interest", they will justify drawing causal inferences "only when causation is assumed in the first place" [63]. Put a bit more charitably, unless there are independent reasons for believing that statistical associations are causal relations, there is no justification, using only these kinds of statistical models, to infer that the statistical associations are causal relations.

For these reasons (and there is no implication intended here that these exhaust the reasons), a second approach for justifying causal inferences, and so warranting the causal claims based on those inferences, has developed. This approach, often adopted independently of the statistically based approach to justifying causal inferences, focuses on identifying and describing the conditions that must be satisfied in order for the belief, that a statistical association between two events is a causal relationship, to be a justified (warranted) belief. Although Susser refers to this as a strategy in which "making inferences about causes" depends on the "subjective judgment" of the person making the judgment [64], this is not an altogether fair characterization. As has already been noted, approaching the problems posed by causal inference using statistical models and estimating causal parameters within those models requires making a variety of assumptions and so inevitably involves "subjective judgment". Subjective judgments are ubiquitous in any account of causal inference, and so is not a characteristic that permits distinguishing formal, statistically based causal inferences from causal inferences based on some other approach. Instead, what distinguishes the conditions-based approach is precisely the idea that a statistical association is a causal relation just in case that association satisfies some set of criteria that is neither reducible to, nor eliminable in favor of the specification of some set of formal statistical models of the statistical association. Thus, Greenland characterizes this approach as one not based "on a formal causal model", and refers to it as the "canonical approach" since it "usually leaves terms like 'cause' and 'effect' as primitives ... around which ... self-evident canons [criteria] are built, much like axioms are built around the primitives of 'set' and 'is an element of' in mathematics" [65]. Historically, the "canonical approach" is evidenced in the 1964 Surgeon General's report on the dangers of smoking. According to the Report:

Statistical methods cannot establish proof of a causal relationship in an association. The causal significance of the association is a matter of judgment which goes beyond any statement of statistical probability [66].

In effect, the Report is stating that no formal statistical modeling of the data can, without additional, non-statistical assumptions, justify drawing a causal inference (and so drawing a warranted causal claim) from any statistical associations that are present. Because of this limitation of statistical modeling, the Report goes on to state that to "judge or evaluate the causal significance of the association between the attribute or agent and disease, or effect upon health, a number of criteria must be utilized, no one of which is an all-sufficient basis for judgment" [66]. The criteria used in the Report were the consistency, strength, specificity, temporal relationship, and coherence of the association.

Following the publication of the Surgeon General's Report, Austin Bradford Hill, in his 1965 Presidential Address to the Section of Occupational Medicine of the Royal Society of Medicine, asked under what circumstances we can justifiably pass from "an observed *association *to a verdict of *causation*" [67]. In answer to this question, Bradford Hill recommended the use of the five criteria present in the Surgeon General's Report, and added four others, viz., biological gradient, plausibility, experiment and analogy [67]. Although he described the circumstances whose presence permitted passing from an observed observation to a verdict of causation as "aspects of [a statistical] association" we should "consider before deciding that the most likely interpretation of it is causation" [67], the resulting nine criteria are now typically referred to as the "Bradford Hill Criteria" for causal inferences. It is true that writers such as Phillips and Goodman object to calling Bradford Hill's aspects of association "criteria", preferring instead the locution "causal considerations" [68], but they also concede that what Bradford Hill proposed is "frequently taught to students in epidemiology and referred to in the literature as 'causal criteria"' [69]. Moreover, while commonly used in epidemiology and the health sciences since 1965 as a "central tool for the epidemiological community in grappling with the broader issues of causal reasoning" [70], the "basic outline of the modern set of criteria has," according to Kaufman and Poole, "evolved little" since their formulation by the Surgeon General's Advisory Committee and Bradford Hill [70].

There are many examples of studies that use the Bradford Hill criteria (or some subset of the criteria) in an attempt to justify causal inferences. One clear and publicly accessible example of their use is on the Website of the SV40 Cancer Foundation. There, Horwin applies "what was published in the peer-reviewed medical literature to the nine Bradford Hill criteria in respect to medulloblastoma and other brain cancers" to demonstrate the causal efficacy of SV40 [71]. In addition, the Environmental Protection Agency's 2005 "Guidelines for Carcinogen Risk Assessment", also publicly accessible, explicitly recommends the use of the Bradford Hill criteria to assess whether an observed statistical association is causal rather than spurious [72]. There are many more examples of applications of the Bradford Hill criteria that appear in academic journals covering a range of disciplines. These examples include, but are not limited to, determining whether chrysotile asbestos causes mesothelioma [73], determining whether second generation antipsychotic drugs cause diabetes [74], evaluating the effects of "environmental carcinogens" [75], evaluating whether abuse experienced as a child or as an adolescent/adult is causally related to urologic symptoms [76], and evaluating causal associations in pharmacovigilance as well as pharmacoepidemiology [77,78]. The Bradford Hill criteria have even been applied to studies in molecular epidemiology [79], as well as to when searching "for the true effectiveness" of dental health care services in facilitating "recovery from an oral health-related decrement in quality of life called 'oral disadvantage due to disease and tissue damage'. [80]" Overall, regardless of the specific discipline in which the study occurred, the most common use of the Bradford Hill criteria when investigating whether a statistical association is a causal relationship (e.g. the statistical association between genital ulcer disease and the transmission of human immunodeficiency virus [81]) is to apply them to evidence presented in reviewed literature [73,74,81-87].

Based on their widespread use, it is not surprising that some form of Bradford Hill's causal criteria are, according to Weed, "arguably the most commonly-used method of interpreting scientific evidence in public health" [88], and that, according to Parascandola, the Bradford Hill criteria are "routinely cited as authoritative statements of the proper method for assessing a body of etiological evidence" [89]. Indeed, Shakir and Layton even go so far as to write that Bradford Hill's Presidential Address, in which the nine criteria ("aspects of association") were identified and described, was one "of the most important papers published in the 20th century with thoughts on the epidemiological basis of disease causation" [77]. Still, just as the popular consent to a belief does not make that belief true, so too, the widespread acceptance and use of Bradford Hill criteria does not entail that their use truly justifies causal inferences. Thus, we need to examine, carefully and critically, the Bradford Hill criteria to determine precisely what their function is, if any, in justifying causal inferences.

The first thing to keep in mind is that 'inference' has at least two meanings that it is important not to conflate. The first meaning of 'inference' is the psychological activity of accepting a conclusion based on one or more other beliefs held to be true. For example, when consumer psychologists study under what circumstances consumers generalize from specific information to general conclusions, or construe specific conclusions from general principles or assumptions [90], they are studying inference as a psychological activity. It is this sense of inference that is important when characterizing rationality [91]. The second meaning of 'inference' is about logical permissibility; it refers to whether one is logically permitted to assert that a particular claim is true because of its evidential relationship to one or more other claims (hypothetically) accepted as true. Here the focus is not on the psychology of people engaged in reasoning, but on the relationship between evidence (claims held true) and a claim asserted to be true. When applying Bradford Hill criteria to causal inferences (inferences having a causal claim as a conclusion), it is the second meaning of 'inference' that is relevant, not the first. In other words, inference, in the context of applications of Bradford Hill criteria, does not refer to the psychological activity of "transitioning" (reasoning) from a set of beliefs to another belief, but instead refers to the kind of evidential relationship that exists between a claim (e.g. a causal claim such as "X causes Y") and the evidence for that claim.

Typically, evidential relationships between evidence held true (the premises) and a claim asserted to be true (the conclusion) because of the evidence are characterized as either deductive or inductive. In the first case, if the deductive relationship is a valid one, then the truth of the evidence guarantees that the asserted claim, the conclusion, is true. Again, it is important to emphasize here that this is a claim about logical implication, not about reasoning. As noted by Harman, it is "an interesting and nontrivial problem to say just how deductions are relevant to reasoning," but it is an interesting and nontrivial problem just because deductive relationships are not instances of reasoning [92]. In the second case, if the inductive relationship is a strong one, then, following Skyrms, "it is improbable, given that the premises [evidence presented in the form of statements] are true, that the conclusion is false" [93]. Thus, in the case of the inductive relationship, the evidence presented by the premises underdetermines the truth-value of the conclusion. Once again, though, this is a claim about the character and limits of logical inference, not reasoning.

To the extent that we are willing to model evidential claims and claims that constitute the conclusions of deductive implications in formal logical systems, it is possible to give system-relative, precise syntactic and semantic characterizations of the concept of deductive validity. For example, suppose that A_1_...A_n-1_, A_n _is a sequence of well-formulated formulae in a formalized logical language L, where A_1_...A_n-1 _are the premises and A_n _is the conclusion. We can then say that A_1_...A_n-1_, A_n _is (syntactically) valid in L "just in case A_n _is derivable from A_1_...A_n-1_, and the axioms of L, if any, by the rules of inference of L" [94]. Analogously, we can say that A_1_...A_n-1_, A_n _is (semantically) valid in L "just in case A_n _is true in all interpretations [models] in which A_1_...A_n-1 _are true" [94]. Of course, this kind of technical sophistication raises an immediate problem if one believes that satisfactions of Bradford Hill criteria are deductively related to a causal claim. The instances of criteria satisfaction, as well as the causal claim functioning as the conclusion, must be "appropriate" instantiations of well-formed formulae in a formalized logical language L. However, except for small, artificially regimented fragments of natural languages, the project of modeling complex natural languages into an underlying formalized logical language (a problem in logic, not linguistics [95]) has met with mixed success and no consensus. The point, then, is that if one holds that satisfactions of the Bradford Hill criteria (validly) deductively support a causal claim, it seems unlikely that it is this highly formalized conception of deductive validity that is at work.

Still, perhaps one could try to use a more informal characterization of a valid deductive inference and say that as long as all the Bradford Hill criteria were satisfied in some acceptable way, they would guarantee the truth of the causal claim. However, by giving up the formalized conception of deductive validity, we have also given up the utility of this more loosely characterized sense of deductive validity. To see why, suppose we let B_1_...B_9 _represent each of the Bradford Hill criteria, and suppose that C represents the causal claim. On the more informally characterized sense of deductive validity, we want to say that on a non-formal construal of the criteria that permits us to determine whether each of the nine criteria are satisfied, and so true, if each of B_1_...B_9 _is true, then C must be true as well. It is not enough to say simply that C is true (as opposed to **must **be true), since C could be true for reasons that have nothing to do with each of or all of B_1_...B_9_. However, what is it about each of or all of B_1_...B_9 _being true that necessitates C being true? It cannot be because of the syntactic characteristics of well-formed formulae in a formalized logical language since we have already given up this characterization of deductive validity. Importantly, it also cannot be because every model in which each of the B's in B_1_...B_9 _is true, is also a model in which C is true, since the specification of models requires adopting the formalized conception of validity [96] that we have given up. Thus, there is no useful sense in which the truth of a causal claim can be "clinched", deductively, by the satisfaction of the Bradford Hill criteria.

Now, implicit in the discussion to this point is the assumption that the relationship between the Bradford Hill criteria and a causal claim is that if the criteria are all satisfied, then the causal claim is true. This is an argument structure known as affirming the antecedent (*modus ponens*), and captures the idea that the satisfaction of the Bradford Hill criteria confirms the truth of the causal claim. However, instead of using this argument structure, we could adopt a broadly Popperian perspective and, instead, use the argument structure of denying the consequent (*modus tollens*) [97]. If we do this, we have moved from a deductivist account of confirmation to a deductivist account of falsification. By doing this, we could say that what matters is not whether the Bradford Hill criteria are satisfied, but whether the criteria are not satisfied. In other words, our argument now has the form that if a particular causal claim, C, is true, then the Bradford Hill criteria, B_1_...B_9_, are satisfied, and if it is not the case that B_1_...B_9 _are satisfied, then C is false. Rather than finding out what causal claims are true, by falsifying the Bradford Hill criteria (i.e., by finding that it is **not **the case that the Bradford Hill criteria are satisfied), we discover which causal claims are false.

However, right away there are problems. First, the expression "it is not the case that B_1_...B_9 _are satisfied" is ambiguous. It could mean either that none of the B_1_...B_9 _are satisfied, or that at least one of the B_1_...B_9 _is not satisfied. The former seems an unlikely interpretation since one of the Bradford Hill criteria is that in a cause-effect relationship, the cause temporally precedes the effect. Arguably, for almost all cases of cause-and-effect relationships in epidemiology, health services research and the social sciences, this will be true [22,88]. Thus, for all but the most extraordinary cases, at least one of the B_1_...B_9 _is satisfied, thereby undermining the deductive inference that the causal claim, C, is false. Second, recall that one of the Bradford Hill criteria is strength of analogy. Analogies are inductive arguments, and so vary along a continuum in terms of their strength [98]. It follows from this that the B in B_1_...B_9 _that corresponds to the Bradford Hill criteria of analogy will never be entirely satisfied (unless the analogy is actually an identity) and never entirely dissatisfied (unless there are absolutely **no **shared properties or characteristics). If we count any degree of satisfaction as sufficient for purposes of claiming that the criterion is satisfied, then we have a problem analogous to that posed by the criterion of the cause preceding the effect. If we try to set some threshold limit for satisfaction, then the assessment of whether the criterion is satisfied seems *ad hoc*.

All this would seem to lead to saying that "it is not the case that B_1_...B_9 _are satisfied" means that there is some proper subset of B_1_...B_9 _none of whose members is satisfied. However, this leads to the possibility of very different assessments of the same causal claim. For example, suppose that the causal claim in question is C, and one person claims that the relevant conditional in the falsificationist inference is "If C then B_1_...B_3_", while another person claims that the relevant conditional is "If C then B_4_...B_9_". Further, suppose that each of B_1_...B_3 _is false while none of B_4_...B_9 _is false. In this case, the first person concludes that the causal claim, C, is false, while the second person claims that there is no justifiable reason to hold that the causal claim is false (and may, in fact, hold the causal claim to be true because it has not been falsified). Although not strictly inconsistent with one another (the failure to falsify a claim does not entail that the claim is true), the two claims are quite different and, at least in a public health context, could lead to the adoption of very different policies. One obvious way to resolve the dispute would be to provide some kind of justification that supports the use of one of the proper subsets of Bradford Hill criteria but not the other. This tact, though, raises its own problems. First, the problem is not simply that we have to choose between two contenders. What we must do is to choose amongst all possible contenders (e.g. there is also the contender of B_3_...B_5_). Second, what kind of justification would suffice for choosing one proper subset of Bradford Hill criteria instead of another? The aim of the Bradford Hill criteria, on the falsificationist deductivist account, was to permit us to exclude causal claims as false. Now, though, it appears that we need criteria for the criteria, and that we need to specify the relationship (possibly deductive, though this seems to raise the same problems all over again) of those new criteria to the Bradford Hill criteria that we want to retain. Thus, treating "it is not the case that B_1_...B_9 _are satisfied" as meaning that there is some proper subset of B_1_...B_9_, none of whose members is satisfied, seems to be no resolution to the problems associated with treating the relationship between the Bradford Hill criteria and a causal claim as one of deductive entailment.

Finally, and more broadly, regardless of the interpretation given to the expression "it is not the case that B_1_...B_9 _are satisfied", there seem to be problems associated with interpreting the criteria themselves since, as Rothman et al. claim, there are ambiguities, fallacies and vagaries in each of the Bradford Hill criteria [99,100]. For example, regarding the criterion of analogy, Rothman et al. write that whatever "insight might be derived from analogy is handicapped by the inventive imagination of scientists who can find analogies everywhere. At best, analogy provides a source of more elaborate hypotheses about the associations under study; absence of such analogies only reflects lack of imagination or experience, not falsity of the hypothesis" [100]. They conclude, based on similar kinds of analyses of the other eight Bradford Hill criteria, that "the standards of epidemiologic evidence offered by Hill are saddled with reservations and exceptions" [100]. When considered *in toto*, these sorts of problems with treating the relationship between a causal claim and satisfactions of the Bradford Hill criteria as either a confirmationist or a falsificationist deductive relationship support the view that we need to find a different account of the relationship.

As noted earlier, the typical division of logical inferences is into deductive and inductive inferences. Thus, because there are good reasons to reject the view that the relationship between satisfactions of the Bradford Hill criteria and the causal claims they purport to justify is a deductive relationship, the obvious conclusion to draw is that the relationship must be inductive. Since strong inductive inferences, in contrast to valid deductive inferences, make it improbable, but not impossible, that the conclusion of an inductive argument is false given that the premises (evidential statements) are true, then understanding the relationship between satisfactions of Bradford Hill criteria and a causal claim seems consonant with what Bradford Hill claimed about the criteria ("aspects of association"). For example, Bradford Hill writes:

What I do not believe – and this has been suggested – is that we can usefully lay down some hard-and-fast rules of evidence that *must *be obeyed before we accept cause and effect. None of my nine viewpoints can bring indisputable evidence for or against the cause-and-effect hypothesis and none can be required as a *sine qua non *[67].

As suggested by this quotation, Bradford Hill did not conceive of the satisfaction of the "aspects of statistical association" as sufficient conditions (singularly or jointly) for justifying a claim that a specific association was a causal relation [101,102]. Moreover, with the possible exception of the temporal priority of a cause to its effect, he did not conceive of the satisfaction of the "aspects of statistical association" as necessary conditions (singularly or jointly) for a specific statistical association being a causal relation. Based on this, it seems reasonable to conclude that Bradford Hill's own understanding of the criteria is consistent with the view that the support their satisfaction offers to a causal claim is something less than that their satisfaction deductively entails the truth of a causal claim. This is certainly consonant with many writers who advocate, use or discuss the Bradford Hill criteria. For example, Russo and Williamson write that "while these criteria were intended as a guide in assessing causality, they do not ensure causality with certainty" [103], while Kundi writes that satisfaction of the Bradford Hill criteria are non-conclusively supportive of a causal claim "but cannot be used to dismiss the assumption of a causal claim" [104]. Similarly, in studies that use Bradford Hill criteria, at least some qualify their conclusions by claiming that the statistical associations are "likely to be causal" [45], that the evidence provided by the criteria's satisfaction underdetermines the truth of the causal conclusion [105], or that satisfaction of the criteria only decreases the likelihood that statistical association is not causal [106]. To sum up, there are good reasons for understanding the satisfaction of Bradford Hill criteria as inductively justifying a causal claim, which amounts to claiming that the criteria, to use Cartwright's useful expression, merely *vouch *for the truth of a causal claim without offering any assurance of its truth [37,107].

Before assessing the use of satisfactions of Bradford Hill criteria as evidence in an inductive inference, we need to be clearer about what it means to call an inference an inductive inference. As Bird notes [108], there are two distinct senses of what it means to be an inductive inference that are often confused. Although both agree that inductive inferences, unlike valid deductive inferences, are ampliative, they differ in their specificity and precision. On the one hand, inductive inferences are those kinds of ampliative inferences in which the premises are specific (usually empirical) statements, and the conclusion is a general statement. For example, although, regrettably, conflating the logical and psychological conceptions of induction, Rothman writes that the "method of induction starts with observations on nature. To the extent that the observations fall into a pattern, the observations are said to induce in the mind of the observer a suggestion of a more general statement about nature" [109]. The classic example of this kind of inductive inference is enumerative induction, which has the general form that from the fact that all *observed *A's are B's, we may infer that it is not probable that all A's (or some percentage of A's larger than the percentage observed) are B's is false. On the other hand, there is a broader meaning of inductive inference. According to this broader meaning, an inductive inference is any logical inference that is not deductively valid inference where, if the inference is a strong one, "it is improbable, given that the premises are true, that the conclusion is false" [93]. There are at least two reasons for preferring the latter to the former meaning of inductive inference. First, not all traditionally acknowledged examples of inductive inference fit the model exemplified by enumerative induction. For example, the inference from the sun having risen every morning in recorded history to the conclusion that the sun will rise tomorrow is an inductive inference from a general premise to a particular conclusion [110]. Second, the broader meaning of inductive inference permits us to separate more clearly the logical sense of inference from the psychological sense of inference. While assertions about inductive inferences express the speaker's beliefs, they are not, as noted, by Maher, "*about *the speaker's beliefs" [111]. Moreover, the broader meaning of inductive inference includes, when attention is restricted to the logical sense of inference, the narrower meaning of inductive inference as an inference from particular premises to a general conclusion. For these reasons, the following analyses use the second, broader meaning of inductive inference.

Let us suppose that B_1_...B_9 _represent the nine Bradford Hill criteria and that C represents a causal conclusion. On the assumption that each of B_1_...B_9 _is satisfied and so true, then B_1_...B_9 _strongly inductively supports C just in case it is improbable that C is false. However, the natural question to ask at this point is whether it is, in fact, true that if each of B_1_...B_9 _is satisfied, and so true, then it is improbable that C is false. This is a form of what is sometimes known as the "problem of induction." More generally, the problem, as has been long recognized, is to state precisely what it is about a set of conditions that guarantees that when those conditions are satisfied, this satisfaction makes it improbable that the associated conclusion is false. If we cannot identify what it is about the conditions that guarantee this result, then there will be no way to distinguish strong inductive inferences from weak inductive inferences. Indeed, it was Hume's inability to identify what it is about what he called the "experimental method" that guaranteed the improbability of inferred conclusions being false that led him to treat the problem of inductive inference as a problem of human psychology. For Hume, there is no logical sense of inductive inference; inductive inferences are all psychological inferences [112,113].

The works of Rudolf Carnap illustrate one approach to making sense of the logical conception of inductive inference. Because, according to Carnap, "the fundamental concept of inductive logic is *probability *[114]", he begins by drawing a distinction between what he calls the logical sense of probability, understood as "degree of confirmation", and the empirical concept of probability (statistical probability), understood as "the relative frequency in the long run of one property with respect to another" [115]. Based on this distinction, Carnap writes that the goal of inductive logic is to "measure the support which the given evidence supplies for the tentatively assumed hypothesis" [115], where the support is formalized in terms of "degree of confirmation", and so, logical probability. In the case of the Bradford Hill criteria, this means that, from the Carnapian point of view, what inductive logic should do is the measure the support that satisfactions of the criteria provide for the causal claim hypothesized as a possibility based on an already identified statistical association. Since the relevant conception of probability is logical probability, to accomplish this task, Carnap believed that it is necessary to characterize inductive logic "like deductive logic ... [as] a branch of semantics. [115]" This understanding of inductive logic raises at least three different problems. First, it requires a precise, "rational reconstruction" of the satisfactions of the Bradford Hill criteria, and the causal conclusion, as appropriate instantiations of well-formed formulae within a logical system where the rules of inductive logical inference are defined. This mirrors the requirement, considered earlier, for treating the relationship between satisfactions of the Bradford Hill criteria and a causal claim as a valid, deductive relationship. Making certain that (claims about) the applications of the Bradford Hill criteria are "appropriate" instantiations of well-formed formulae in the theory of inductive inference is a necessary condition for validating, within the inductive theory, the claim that satisfactions of the Bradford Hill criteria inductively support the inferred conclusion [114]. As such, the same kinds of problems associated with the identification and translation of natural language sentences into well-formed formulae in the case of treating the relationship between satisfactions of the Bradford Hill criteria and a causal claim as a deductive relationship occur here as well.

Second, even assuming that there is an acceptable solution to the problem of providing the appropriate rational reconstructions, there is still the problem of validating the inductive inference rules that constitute the system of inductive logic into which the satisfactions of the Bradford Hill criteria and conclusion have been translated. This is the problem of the justification of induction. Although there are many formulations of the problem, one way to formulate it is to take advantage of Carnap's claim that inductive logic, like deductive logic, is a branch of semantics. Thus, if A_1_...A_n-1_, A_n _is a sequence of well-formulated formulae in a formalized logical language L, where A_1_...A_n-1 _are the premises and A_n _is the conclusion, then A_1_...A_n-1_, A_n _is (semantically) inductively strong in L just in case it is improbable that A_n _is false in all interpretations [models] in which A_1_...A_n-1 _are true. The problem, then, is whether there are any inductive inference rules whose adoption is consistent with the semantic conception of an inductively strong argument [116]. It is true that one obvious kind of response to this would be to say that if an inference rule, R, is, in all *observed *instances of application, consistent with the semantic conception of an inductively strong argument, then we are justified in using the inference rule. However, as should be obvious from this formulation, this response is tantamount to using a kind of inductive inference to justify the inference rule R. In this case, though, the problem remerges when asked to justify this additional inference rule, and an infinite explanatory regress threatens the entire account. Although there are other approaches to justifying induction (e.g. the pragmatic justification originated by Reichenbach [117] and the analytic justification suggested by Harré [118]), "none has received widespread acceptance. [119]"

The third problem is one that, following a suggestion by Hempel, we might call "the problem of desiderata" [120]. This is the problem that in any inductive determination of the degree of confirmation conferred on a conclusion from premises assumed to be true, it is not enough to take into account only the information provided by the premises. Hempel frames the problem by asking the following question:

On the basis of different sets of statements that we consider as true, a given hypothesis *h *... can be assigned quite different probabilities; which of these, if any, is to count as a guide in forming our beliefs concerning the truth of *h *and in making decisions whose outcome depend on whether *h *is true? [121]"

According to both Hempel and Carnap, to answer this question requires the adoption of a principle known as "the requirement of total evidence." As noted by Carnap, the requirement of total evidence says that in any inductive inference, "we have to take as evidence ... the *total evidence *available to a person in question at the time in question, that is to say, his total knowledge of the results of his observations" [122]. The requirement of total evidence is not a requirement of the formal inductive system of logic but is, instead, "a maxim for the *application *of inductive logic" [123]. While it may seem simple enough to incorporate this requirement, its adoption (even ignoring the problems of formalization already faced by treating applications of Bradford Hill criteria to support a causal claim as inductive inferences) has at least two unwelcome consequences. First, it means that all inductive inferences are relative to the knowledge possessed by the person making the inferences. Thus, all assessments of inductive inferential strength require a full accounting of the relevant background information, and consequently entail that we need some means of assessing amounts and kinds of information. Second, and more worrisome, the requirement seems to lead to the "new riddle of induction" identified and described by Nelson Goodman [124]. The problem, put briefly, is that once the need for such information is conceded, no matter what additional information is provided, that evidence, together with the evidence provided by the other statements assumed true, from which an inductive inference to a conclusion is drawn, underdetermines what conclusion it is permissible to draw. The threat, then, is that any set of inductive inferential rules strong enough to justify claiming that a statistical association is a causal relation will permit too much. There is no principled way to say that the application of a set of inductive inference rules, together with an assumption that a set of premises (e.g. applications of Bradford Hill criteria) are true and a specification of the "total evidence" available, will justify inductively inferring a single conclusion as opposed to a myriad of other conclusions [125].

Still, perhaps we can successfully accomplish in the case of inductive inferences what we could not in the case of deductive inferences. In particular, maybe we can weaken (make less formal) the characterization of what a strong inductive inference is in a way that permits us to use satisfactions of the Bradford Hill criteria to justify, in some looser inductive sense, a causal claim. One possibility along these lines is to say that although they are not rigid criteria whose satisfaction is required for making a justified causal inference, applications of the criteria "still give positive support to inferences about causality" [126], and one can compare the results of commensurate applications of the criteria to one another. There are two key ideas at work here. The first is that while no satisfactions of any of the criteria are, singularly or jointly, necessary or sufficient for justifying the claim that a statistical association is a causal claim, the satisfactions of one or more of the criteria provide at least some informal inductive support to the claim that a statistical association is a causal relation. The second key idea is that there is no specific requirement for "rational reconstruction" of the satisfactions of the Bradford Hill criteria or the causal conclusion into a formalized language within which precise characterizations of the inductive inferences exist. Instead, there is a much looser idea at work. Regardless of how we assess whether or not, and to what degree the Bradford Hill criteria are satisfied, as long as there are consistent assessments of applications of the Bradford Hill criteria we can create ordinal rankings of sets of assessments. For example, on the assumption that the strength of a dose-response is an indicator of the presence and strength of a biological gradient, then in the case where there are two statistical associations to the same event, the statistical association having the stronger dose-response provides the greater positive support to its claim that the statistical association is a causal relation [74,87,126-129].

While this avoids some of the problems associated with a more formal characterization of inductive inferences and inductive inferential rules, there are at least three problems with this interpretation of the inductive support provided by satisfactions of the Bradford Hill criteria. First, to the degree that Rothman et al. are correct that "the standards of epidemiologic evidence offered by Hill are saddled with reservations and exceptions" [100], it will be, at best, difficult to quantify the satisfactions of the criteria to assess degrees of confirmation. Without the ability to quantify the satisfactions of the criteria, the only reference cases against which it seems possible to measure the degree of confirmation are the null case, where no criteria are satisfied, or the singleton case where the one possible *sine qua non *criterion, temporal priority, is satisfied. Although some writers believe that "it is relatively straightforward to describe the conditions" under which the criteria are "clearly not satisfied" [22], using the null case to make comparisons permits too much. The comparison would lead to claiming that any satisfaction of one or more of the criteria is evidence of a causal connection, without permitting any comparison among the cases in which one or more of the same criteria are satisfied. For example, suppose that there are three statistical associations, where commensurate applications of the Bradford Hill criteria to all three results in saying that the first two associations satisfy the same five criteria while the third satisfies only four of the five criteria satisfied by the first two. What can we conclude? If there is no way to quantify the satisfactions of the Bradford Hill criteria, all we can conclude is that the inferences that the first two statistical associations are causal relations are stronger than the inference that the third statistical association is a causal relation. There is, though, no way to make any comparative assessment of the first two statistical associations. The ordinal ranking of satisfactions of the Bradford Hill criteria, in this case, seems too coarse grained to be of much practical value.

The second problem is that even if we can assess degrees of confirmation in a manner that permits a more fine-grained ordinal ranking (and so avoiding the first problem), all causal claims will be relative to other causal claims for which one has good reasons for believing that they have less confirmation. Causal claims are never claims *simpliciter*, but rather are always claims relative to one or more other possible contenders. Using causal criteria to assess whether a statistical association is a causal relationship is, to vary a remark by Rosenberg, "always a comparative affair" [130]. It only makes sense to say that a particular causal claim, C, "is more or less well confirmed by the evidence" relative to the criteria than is causal claim C*, not that C is confirmed, relative to the causal criteria, "in any absolute sense". Thus, imagine that one wonders whether a particular factor (or event), X, that is statistically associated with another factor (or event), Y, is a cause of Y. On this interpretation of the Bradford Hill criteria, the answer is never "yes" or "no", but only "yes" or "no" relative to other possible causes of Y. For example, suppose that the probability of X being a cause of Y, given some measure of the satisfaction of the Bradford Hill criteria, is greater than the probability of some other X* being a cause of Y, given some commensurate measure of the satisfaction of the criteria. It follows from this that we can say that, compared to X*, we are justified in asserting that X is the cause of Y. However, it is important to recognize the limits of this kind of claim. While it may seem that we are led to say that X is the cause of Y while X* is not, that is not correct. Instead, on the assumption that the probability assessment can be made, the most that we can assert is that the causal influence of X on Y is greater than the causal influence of X* on Y. On this account, we can rule out X* having a causal influence on Y only if X* satisfies none of the causal criteria we use to make the causal claim. Thus, except for the limiting case in which none of the criteria is satisfied, the conclusion appears to be that all statistical associations that satisfy Bradford Hill criteria are, to a greater or lesser extent, causal relationships. From the worry of not being able to identify any causal relations, we have slipped to the other extreme of finding too many causal relations; all statistical associations are causal relations, though of varying degree.

The third problem is an extension of the second problem. Suppose that B_1_...B_9 _refer to each of the nine Bradford Hill criteria. Moreover, suppose that we have a statistical association between X and Y, and so wonder whether the claim that X causes Y is justified. To take a simple example, suppose that we know that smoking is statistically associated with cancer, and we wonder whether smoking causes cancer. On the present proposal, what we would do, presumably, is to examine whether the relationship between smoking and cancer satisfies the Bradford Hill criteria. Thus, we could examine how plausible it is to suppose that there is a biological relation relationship between smoking and the cancer in question, we could examine whether the relationship between smoking and cancer has been "repeatedly observed by different persons, in different places, circumstances and times" [67], and so forth. As Weed notes, in cancer epidemiology, the most likely choice of Bradford Hill criteria to use are "consistency, strength, dose response and biological plausibility, leaving behind coherence, specificity, analogy and (interestingly) temporality" [131]. Of course, even by examining all these satisfactions of the Bradford Hill criteria, nothing immediately follows. Because, on this interpretation of the inductive support that satisfactions of Bradford Hill criteria give to a causal claim, assessments of whether a statistical association is a causal relationship are always relative to alternative assessments, we need additional possible causal claims against which to assess the current application of the criteria. What other possible claims should we consider?

One possibility is to say that we should compare the current causal claim against the claim that no causal relationship between smoking and cancer is present. Recall, though, that we make applications of the Bradford Hill criteria only to existing (recognized) statistical associations. Therefore, since the claim that no causal relationship between smoking and cancer is present is, in the limiting case, the claim that there is no statistical association between smoking and cancer, it follows that the limiting case is, *de facto*, ruled out by the presence of the statistical association. This means that we still need another statistical association involving cancer as a "cause" to which we can apply the Bradford Hill criteria and compare the results of those applications to the application of the criteria to the statistical association of smoking and cancer. Since all smoking is an activity associated with many other activities of life, then the obvious choice is to examine whether there is a statistical association between one or more of those other activities of life and cancer. If so, then we can apply the Bradford Hill criteria to those other associations and thus be in a position to make the kind of comparative assessment required by this understanding of the role of the Bradford Hill criteria. It is precisely here that the problem occurs. There is going to be a very large number of statistical associations that we could subject to evaluation by use of the Bradford Hill criteria. Some, such as drinking coffee or consuming alcoholic beverages, present themselves as obvious candidates, while others, such as waking up in the morning, seem to be rather silly. Curiously, it is the silly possibilities that pose the problem. The statistical association between waking up in the morning and cancer may make it a silly candidate for applications of the Bradford Hill criteria to form the appropriate contrasts, but what makes it silly? One might say that what makes it silly is the strength of the statistical association, but of course, this is itself one of the Bradford Hill criteria, and so it follows that this method of demarcation is using one of Bradford Hill criteria to rule out applications of the other criteria.

The question now shifts to what it is that justifies *this *use of the Bradford Hill criterion (the criterion of statistical strength) as opposed some different criterion or set of criteria. The problem is analogous to the "problem of induction" raised earlier. Either we have some other criteria though whose use we justify applying the full range of Bradford Hill criteria to a statistical association, or we do not. If we do, then we have the problem of justifying the application of these new criteria, which seems to threaten the same kind of explanatory regress considered earlier. If we lack some other criteria, then either the choice to take only some and not all statistical associations seriously is *ad hoc*, or else, to be consistent, we need to evaluate all the statistical associations. In the former case, there is no basis for resolving disagreements between choices of which statistical associations to subject to evaluation by applications of the Bradford Hill criteria. You choose one set of statistical associations and I choose another, and (apart from a way of adjudicating different theories incorporating different causal claims) that is the end of the matter. Although this state of affairs appears to reflect Susser's observation that in the case of judgments about causality, "there are no absolute rules, and different workers often come to conflicting conclusions" [64], it is difficult to understand why, even if true in practice, one would embrace this as a welcome entailment of a theory of causal inference. In the latter case, the requirement to test all the statistical associations is, except for very narrowly defined and artificial cases, practically impossible.

Suppose, though, that we somehow agree (and that our agreement is, in some sense or another, "justified") on a set of alternative statistical associations to which we will apply the Bradford Hill criteria. To keep matters simple, imagine that we have agreed that there are only two statistical associations to assess, and that X-Y is the first statistical association while X*-Y is the second statistical association. Since we have agreed to assess both, we apply the Bradford Hill criteria to the two associations (where the applications are commensurate to one another) and report the results. In the first case, by applying the criteria we discover that we have measures for six of the nine criteria, while in the second case, we have measures for only five of the nine criteria. In addition, we discover that there is information on an application of at least one criterion in each of the two sets for which information in the other set does not exist. Using B_1_...B_9 _to represent the nine criteria (with no correspondence to the order in Bradford Hill's presentation intended), we have information on the satisfaction of B_1_...B_6 _in the first case, while in the second case we have information on the satisfaction of B_3_...B_7_. The problem is that because different sets of Bradford Hill criteria are satisfied in the two cases, any ordinal comparison of the two applications can only be on the overlapping criteria. That may not seem so problematic in this case, but suppose that we have a third statistical association, X**-Y to which we can apply (for whatever reason) only one of the Bradford Hill criteria. In this case, to use the ordinal metric presupposed by the interpretation of the inductive character of the Bradford Hill criteria we are examining requires that we can only compare the three statistical relationships based on the application of the single Bradford Hill criterion. Notice that while some "weight of evidence" methodologies suggest otherwise [132], it will not do to say that the inability to apply a Bradford Hill criterion is the same as saying that the Bradford Hill criterion is not satisfied. After all, counterfactually, it might be true that if the criterion had been applied in one case (say the case of X-R) it would have had a higher degree of satisfaction that the degree of satisfaction in the case in which it was, in fact, applied (say X*-R). This means that when assessing applications of Bradford Hill criteria to (alternative) statistical associations, we have two options. Either we must use only those criteria applied commensurably applied to all the statistical associations, or we need some way to make assessments about the relative importance of the criteria so that having information about the satisfaction of some counts for more than lack of information about others. In the first case, we could imagine that although forced to use only one criterion, the statistical association actually strongly satisfied the other criteria, but that this was not information we could justifiably use in making the comparative assessment of statistical associations. In the second case, what Weed refers to as the problem of the "selection and prioritization of the criteria" [133], we are back to the problem of needing some additional criteria to assess the relative value of the various Bradford Hill criteria used in making an assessment about a causal claim. For reasons adduced earlier, this seems to lead once again to an explanatory regress.

At this point, we seem led to the conclusion that because there are so many difficulties associated with the use of Bradford Hill criteria, we are justified in expunging their use entirely when assessing whether there is sufficient justification to claim that a statistical association is a causal relation. Regardless of whether the causal inferences based on satisfactions of Bradford Hill criteria are deductive inference or inductive inferences, there are problems that undermine their use in justifying the claim that a statistical association is a causal relation. However, for the supporters and advocates of the Bradford Hill criteria, the situation is not so bleak as is suggested by the foregoing analyses. Recall that Bradford Hill never referred to the "causal criteria" as "criteria" but, instead, referred to them as "aspects of association", "features of consideration" and "viewpoints" [67]. Moreover, as noted earlier, writers such as Philips and Goodman [68,69] go to some pains to point out that the "aspects of association" that we have been referring to as causal criteria "clearly do not meet usual definitions of criteria" [68]. According to Bradford Hill, the value of the "criteria" is that their satisfaction can, "with greater or lesser strength ... help us make up our minds on the fundamental question – is there any other way of explaining the set of facts before us, is there any other answer equally, or more likely than cause and effect? [67]" One way to interpret this claim that significantly weakens the "testing" role of the criteria is that while satisfactions of the criteria are neither necessary nor sufficient conditions for justifying claims that statistical associations are causal relations, they are, nevertheless, good "guidelines" or "rules of thumb" for how we should exercise caution when making causal claims. When inferring a causal relation from a statistical association, we should always keep the Bradford Hill criteria in mind and be conservative in the inferences we accept. On this interpretation, the role of the criteria is not to justify causal inferences, but, instead, to provide some "aids to thought", as Doll puts it [127], to follow whenever we use some other (still undecided method or methods) for justifying causal inferences.

The obvious problem that this interpretation seems to face is that if satisfactions of the criteria are neither necessary nor sufficient for justifiably claiming that a statistical association is a causal relation, then they are neither necessary nor sufficient as recommendations for *how *one should be cautious when making causal inferences. To take a simple example, suppose that someone decides to investigate whether a statistical association is a causal relation and, knowing the Bradford Hill criteria, we caution the person about to conduct the investigation to keep the criterion of constancy in mind when making any causal inferences from the statistical association. The person about to conduct the investigation might very well be puzzled by this and ask both how he or she should take consistency into account when considering the causal inference, and, even more generally, why consistency should be taken into account. In answering the first question, perhaps we should remember the concerns and criticisms of Rothman et al. about the Bradford criteria being "saddled with reservations and exceptions" [100]. If correct, then there is no simple, unequivocal answer to this question. Other than suggesting that the person look for instances of the statistical association in a variety of different conditions, it is not clear what can be said. While this may be helpful in some very general way, this kind of general caution is certainly not unique to the Bradford Hill criteria. The problem posed by the second question is even more severe. Since consistency is not a necessary condition for a statistical association to be a causal relation, then its absence, by itself, cannot undermine the person's causal claim. Moreover, since consistency is not a sufficient condition for a statistical association to be a causal relation, then its presence, by itself, is no guarantee that the statistical association is a causal relation. However, it is really more than this. Presumably, the idea behind treating the Bradford Hill criteria as "aids to thought" or "useful guidelines" is that their use will somehow contribute to an increased likelihood that a causal inference is a justified causal inference. The question, though, is how we are to understand this if the applications of the criteria are not themselves part of the inferential justification. It may be true that satisfaction of the criteria results in a greater likelihood that one will correctly apply whatever method one chooses to use to justify causal inferences. Unfortunately, this does not seem like a plausible interpretation. On the one hand, the criteria do not seem to be about the use of methods, but rather about statistical associations. On the other hand, even if they are "aids to thought" whose usefulness comes from constraints they place on applications of some chosen method for making causal inferences, why suppose that the method for which the Bradford Hill criteria are constraints is the (or at least a) proper method? If the method for which the Bradford Hill criteria are constraints is the "correct" method because the Bradford Hill criteria guide that inferential method "in the right way" in identifying causal relations, then, in reality, the Bradford Hill criteria are themselves criteria for making justified inferences, even though they are not the "final" criteria. Here, though, we are back to trying to make some sense of how they can serve this function in light of all the problems associated with linking them to either deductive or inductive inferences. If there is no independent reason for thinking that the method for which the Bradford Hill criteria provide constraints is the appropriate method for identifying which statistical associations are causal relations, then the Bradford Hill criteria have no utility in the project of justified causal inferences. If, though, there are independent reasons for accepting the method for which the Bradford Hill criteria provide constraints, then it is not clear what kind of constraints the Bradford Hill criteria provide. It would seem that applications of the Bradford Hill criteria are, in this case, independent of the chosen method for justifying causal inferences, and so provide no real constraints at all. Thus, either the criteria have very little or no use as meta-methodological criteria, or their use presupposes that they really are, in some way or another, criteria whose use will provide some kind of justification for causal inferences.

At this point, let us backtrack a bit. Suppose that we do concede that even as aids to thought, satisfactions of the Bradford Hill criteria do, in some sense, justify causal inferences and the causal conclusions of those inferences. The objection to this was that the foregoing analyses have demonstrated that there are many difficulties associated with using the criteria, regardless of whether we look at their possible role in deductive or inductive inferences. However, what is important to bring out is an implicit assumption at work in this objection. The implicit assumption is that all logical inferences are either deductive or inductive (or some combination), and that this dichotomy is an exhaustive one. It is certainly true, as remarked earlier, that this is a traditional and widely held view about the nature and character of logical inferences. As it happens, though, the assumption does not appear to be true. Having its roots in C.S. Peirce's account of abduction (or what he later called retroduction), there is a third kind of logical inference that, since the middle 1960s, has played "an enormous role in many philosophical arguments and, according to its defenders, an essential role in scientific and common-sense reasoning" [134]. This third kind of logical inference is called "inference to the best explanation" [135,136], and it is here, I believe, that we can find a defensible role for the Bradford Hill criteria.

As noted by Thagard, in "his writings before 1890, Peirce classified arguments into three types: deduction, induction, and hypothesis" [137]. However, by the early years of the twentieth century, Peirce had substituted "abduction" for "Hypothesis", and would later substitute "retroduction" for "abduction". For example, in an April 1903 lecture delivered at Harvard University, Peirce said that there are three different kinds of reasoning – "Abduction, Induction, and Deduction" [138]. For Peirce, deductive reasoning "is the only necessary reasoning" [138] and proves that something *must *be" [139], and inductive reasoning "is the experimental testing of a theory" [138] that "consists in starting from a theory, deducing from it predictions of phenomena, and observing those phenomena in order to see *how nearly *they agree with the theory" [139]. In contrast to both deduction and induction, abduction "consists in studying facts and devising a theory to explain them, [138]" and in this way, "is the process of forming an explanatory hypothesis" [139]. Thus, for Peirce abductive reasoning is a kind of logical inference that begins with the available facts "without, at the outset, having any particular theory in view, though it is motivated by the feeling that a theory is needed to explain" the facts [140], and discovering a conjecture (hypothesis) "that furnishes a possible Explanation" [141].

In 1965, Gilbert Harman introduced the expression "inference to the best explanation" and wrote that "'The inference to the best explanation' corresponds to what others have called 'abduction"' [135]. According to Harman, in making an inference to the best explanation, "one infers, from the fact that a certain hypothesis would explain the evidence, to the truth of that hypothesis" [135]. Of course, it is likely that there will be a number of hypotheses that, to one degree or another, "explain" the evidence. What inference to the best explanation provides is a method wherein by "starting out with a set of data", we are justified in inferring what hypothesis to take seriously as a starting point for further investigations on the grounds that the hypothesis is the best (in some, to this point, undefined sense of "best") hypothesis that explains the data [142]. Sometimes, the method of inference to the best explanation is expressed counterfactually. For example, Lipton writes that we should understand inference to the best explanation as an inference in which given "our data and our background beliefs, we infer what would, if true, provide the best of competing explanations we can generate of those data" [136]. The importance of the counterfactual formulation of inference to the best explanation is that it presents the hypothetical character of the conclusion of the inference. In inference to the best explanation, what we get is a hypothetical truth rather than a conclusion guaranteed true or confirmed improbable to be false. This concurs with Peirce's claim that abduction "does not afford security" [141] and that its purpose is to create a hypothesis, explaining the data, which we must then test by the appropriate deductive and inductive inferences.

Although there is debate about whether contemporary characterizations of inference to the best explanation (IBE) fully and accurately capture the view of abduction (retroduction) to which Peirce finally came [143,144], there are three important characteristics of most contemporary formulations of IBE that are largely shared with various remarks in Peirce's writing. First, while the traditional characterizations of deductive and inductive inferences take place independently of characterizations of what constitutes an explanation, there is a combination of inference and explanation in IBE. As Lipton writes, far "from explanation only coming on the scene after the inferential work is done, the core idea of Inference to the Best Explanation is that explanatory considerations are a guide to inference" [136]. In a similar vein, Douven writes that advocates of IBE "all share the conviction that explanatory considerations have confirmation-theoretical import" [145]. The second characteristic of IBE shared with Peirce's conception of abduction/retroduction is that IBE is a logical inference. In the context of examining the role of the Bradford Hill criteria, this is an especially important point. The dilemma presented by the earlier analysis was that either we understand applications of Bradford Hill criteria in their role as premises in deductive or inductive causal inferences, or we understand applications of Bradford Hill criteria as having no direct role in causal inferences. Both horns of the dilemma seem to lead to unacceptable problems, but in linking applications of the Bradford Hill criteria to IBE, we grasp the dilemma by the first horn, and attempt to defuse the dilemma by identifying a role for applications of the Bradford Hill criteria in a different kind of causal inference. The third characteristic, related to the tie between inference and explanation in IBE, is that IBE is not a "logic of proof" in the sense that deductive and inductive inferences are logics of proof, but is instead a "logic of discovery" [146-149]. What this means is that the explanatory character of IBE entails that the inference does not simply restate information already present in the data from which it starts (as in deduction) or try to use information already present in the data to confirm the low probability that a conclusion is false (as in induction). Instead, in IBE the data provides the context for making a logical, albeit non-deductive and non-inductive, inference to a hypothesis that (best) explains the facts. In this sense, IBE "discovers" the hypothesis that best explains the data. Thus, IBE rejects Popper's claim that "conceiving or inventing a theory" does not call for "a logical analysis" and that there "is no such thing as a logical method of having new ideas, or a logical reconstruction of this process" [146]. Using a distinction drawn by Hanson, we can make the point by saying that whereas both inductive and deductive inferences provide justification for a hypothesis, IBE provides good reasons for "suggesting" a hypothesis, whose justification (in the former sense of deductive or inductive inferential inquiry) we ought to undertake [147,149]. Admittedly, there is some tension between advocates of IBE who insist that IBE provides reason for believing that the hypotheses resulting from applications of IBE to data are true [134,135,142] and those who believe that while the hypotheses have explanatory virtues we should refrain from calling them "true" [144]. However, the counterfactual formulation, that inference to the best explanation results in a hypothesis that, if true, would provide the best explanation, is the "middle" position capturing the important elements of both sides in the debate. Moreover, this interpretation of IBE seems best suited to distinguish clearly IBE, as a logical inference, from both deductive and inductive inferences where the (necessary or probable) truth or falsity of the conclusion is an important characteristic of the inference. Consequently, in the discussions and analyses that follow, the form of IBE used is one that incorporates the counterfactual truth-value characterization of the conclusion of the inference.

Before fleshing out some of the details, it is worth noticing that understanding the role of satisfactions of the Bradford Hill criteria in this way – as the data used in IBE – seems to sit well with at least some accounts of the role of the Bradford Hill criteria in epidemiology and health services research. For example, Kaufman and Poole write that lists of causal criteria, such as the Bradford Hill criteria, have emerged "as informal test of whether alternative explanations (e.g. confounding) are likely to exist for the hypothesis of causality" [70]. Put into the language of IBE, applications of the Bradford Hill criteria to data lead to the discovery of the most plausible (hypothetical) explanation of an observed statistical association. In a similar vein, Phillips and Goodman suggest that the Bradford Hill criteria (which they insist are not criteria at all) function informally to introduce "common sense" into the search for what causal claims to accept [68]. If "common sense" is understood as a kind of process of discovering possibilities and weeding them out, a view of common sense that, as noted by Höfler, is consistent with the philosophical tradition [150], then this view is, in important respects, similar to the view in which satisfactions of the Bradford Hill criteria play a role in IBE. In his discussion of the precautionary principle and public health, Weed makes a comment that seems to suggest that he too might be amenable to linking satisfactions of the Bradford Hill criteria to IBE. Weed writes that causal criteria are "the most commonly-used method of interpreting scientific evidence in public health", and that the criteria "are 'applied' to the available evidence after it has been collected and summarized in a systematic narrative review" [88]. If we focus on the ideas of interpretation and applications to available data, then this view, in its broad outlines, seems consonant with the idea that, in IBE, the inference is an instance of both a logic of justification (proof) and a logic of discovery. Finally, even Bradford Hill seems to have had something like the IBE role of the criteria in mind when writing about them in his Presidential Address. What Bradford Hill claimed in that address is that the satisfactions of the criteria can help us in making up our minds about the "fundamental question – is there any other way of explaining the set of facts before us, is there any other answer equally, or more, likely than cause an effect? [67]" Here, what Bradford Hill has done is to link explicitly the kind of inference supported by satisfactions of the criteria with "explaining the set of facts before us", which is precisely the kind of link IBE makes.

What, then, does it mean to place satisfactions of the Bradford Hill criteria in the framework of IBE? There are at least three important consequences of such a placement. First, and foremost, it means that satisfactions of the Bradford Hill criteria do not "justify" causal claims in the traditional sense of "justify"; satisfactions of the Bradford Hill criteria neither guarantee the truth of a causal conclusion nor make it improbable that a causal conclusion is false. It follows that studies claiming to apply "the criteria proposed by Bradford-Hill to establish causality between associated phenomena" [151] or that satisfactions of the Bradford Hill criteria "operationally" justify the existence of a causal relation [152], have seriously misunderstood the role that satisfactions of the Bradford Hill criteria play relative to causal claims. Within an IBE framework, satisfactions of Bradford Hill criteria do not justify asserting that a causal claim is true. Satisfactions of the Bradford Hill criteria do not provide "a useful tool for the assessment of biomedical causation" [153], and they do not confirm the causal efficacy of an agent (such as cancer) in the emergence of one of more symptoms [86]. Put more generally, causal criteria, within an IBE framework, are not, as Susser suggests, criteria in the "pragmatic inductive/deductive approach" whose function is to "guide the evaluation of evidence about cause" [154]. The mistake here, from the point of view of IBE, is that these claims are attempting to place satisfactions of Bradford Hill criteria in deductive or, more likely, inductive inferences. When used in IBE, applications of Bradford Hill criteria lead to the discovery of explanatory hypotheses whose explanatory power, if true, is what justifies their role as hypotheses from which further (deductive and inductive) investigations should proceed.

Even more cautious claims about the role of Bradford Hill criteria, such as that their satisfaction permits determining whether statistical associations between exposures and outcomes "are likely to be causal" [45], or that the use of the criteria is useful in reviewing the evidence in support of a causal claim [6], are likely inconsistent with the IBE understanding. Although not explicitly stated, such studies seem to make one of two (sometimes both) underlying assumptions. The first assumption is that satisfaction, to some degree, of one or more of the Bradford Hill criteria confirms the claim that a statistical association is a causal relation, while the second assumption is that the failure of those criteria to be falsified gives some reason for accepting that a statistical association is a causal relation. This contrasts with the IBE framework in which satisfactions of the Bradford Hill criteria both identify a hypothesis about a statistical association, and justify claiming that the hypothesis that the statistical association is a causal relation is, if true, the hypothesis that best explains the available data. Steinberg and Goodwin appear to come close to this view of the Bradford Hill criteria. They write that their study about alcohol and breast cancer reviewed "the available evidence regarding the association of alcohol with breast cancer" and then applied the Bradford Hill criteria to the data "to examine the existence and nature of the association of alcohol with breast cancer risk" [87]. If we replace 'examine' with 'discover', and equate discovering the nature of an association with discovering whether treating a statistical association as a causal relation is the best explanation of the statistical association, then we have something reasonably close to the idea of applying the Bradford Hill criteria in an IBE framework.

A second implication of placing the Bradford Hill criteria in an IBE framework is that the relevant inference, with the conclusion that the best explanation for a statistical association is that it is a causal relation, must begin with a body of facts (data) [142]. This is at least superficially consistent with Weed's claims that the "practice of causal inference requires a body of evidence" [155], and, with some possible qualification depending on what Weed means by "collected and summarized", that the criteria "are "applied" to the available evidence after it has been collected and summarized in a systematic narrative review" [88]. Moreover, it seems to accord well with Susser's claim that judgments about the presence (or absence) of causal relations are "reached by weighing the available evidence" [64], and with studies that apply Bradford Hill criteria to collected evidence presented in reviewed literature [73,74,81-87]. The important point here is that the causal claim that is the conclusion of IBE is neither a deductive nor an inductive inference from this data, but is rather an inference in the sense that it is an explanatory claim that, if true, makes the greatest sense of the data. Put a bit differently, the hypothesis generated by IBE is "justified precisely to the extent that it is shown to have explanatory power" [156], and that explanatory power is what is revealed by the satisfactions of the Bradford Hill criteria when applied to the available data.

To reiterate though, one cannot conclude that a causal claim inferentially supported by satisfactions within an IBE framework is a true causal claim or that it is improbable that the conclusion is false. What IBE permits is only the conclusion that the hypothesis that the statistical association is a causal relation is the best possible explanation, given the satisfactions of the Bradford Hill criteria by the data. What the satisfactions of the Bradford Hill criteria do is not make the causal claim true, but instead, justify the claim that the causal claim is the one that would, if true, be the most explanatory in light of the data to which the criteria were applied and the satisfactions of the criteria [136]. IBE, like Peirceian abduction from which it comes, is, in the case of causal inference, the process of adopting a causal claim "on probation". As noted by Curd, this adoption "does not mean accepting the hypothesis [causal claim] as true, or even as inductively probable, but regarding the hypothesis as a workable conjecture, a hopeful suggestion which is worth taking seriously enough to submit to a detailed exploration and testing. [157]" Contrary to Potischman and Weed, this means that even if all the Bradford Hill criteria were applied to the data and all the criteria were, to a greater or lesser degree, satisfied, nothing would follow about whether we would be in a "strong position to make a public health recommendation, as long as other (e.g. ethical) considerations were also met" [105]. This sort of claim conflates the function of IBE with induction. Unlike Harman's view of IBE according to which all inductive inferences are subsumable under the umbrella of IBE [135], the view I am presenting in this paper is that IBE is distinct from inductive inferences. On the other side of the inductive-deductive dichotomy, it is also a mistake to claim that "causal criteria can be used to critically test – through refutation and prediction – causal hypotheses of necessary causes" [44]. This conflates the function of IBE with deduction. The only logically permitted conclusion, within the IBE framework, is that we have good reason for taking seriously the hypothesis that the statistical association is a causal relation. This does not make the conclusion true or likely, or improbable to be false; it only means that it is a hypothesis that we now need to investigate further to determine whether the statistical association really is a causal relation and what causal effect, if any, there is.

The third important consequence of placing the Bradford Hill criteria in an IBE framework is that the relation of satisfactions of the criteria to the hypothesized causal claim is not a formal one. In contrast with deductive inferences and the ideals of inductive inferences, there are no formal rules of IBE. As Hanson notes, for Peirce, one of the forerunners to Hanson's "logic of discovery" and IBE, there is no "manual", no formalized set of rules, to "help scientists make discoveries" about the hypotheses that best explained the data [147]. Instead, the rules of IBE are best thought of as strategies [158] to accomplish a particular goal, viz., the goal of making explanatory sense out of the data in question, where the "explanatory sense" in question means explanations within a cause-and-effect framework. In this respect, the inferences in IBE are somewhat different from the way that Hanson characterized inferences in his "logic of discovery". As Gutting notes [159], one of the principal objections to Hanson's "logic of discovery" as well as why, for Gutting, Hanson's "analysis remains unfruitful" is that he conceived of its inferences having a logical form in the same sense that deductive and inductive inferences have a logical form. By characterizing the rules of IBE (instantiated by the Bradford Hill criteria) as strategies (regulative principles), one avoids the problems associated with treating them as formal, logical rules of inference, while, using language from Simon, retaining their "logical" status as "normative standards for judging the process used to discover" the best explanatory hypothesis [148].

It is here that one's assumptions about the "nature" of causes impacts the kinds of acceptable inferences to the best explanation. If one, *pace *Cartwright, believes that "there is an untold variety of causal relations" [107], then there will not be a single answer to what the "best" causal explanation is. The answer will vary with the kind of cause (or causes) in which one is interested. This fits well with a claim already attributed to Weed that, in cancer epidemiology, the most likely choices of Bradford Hill criteria to use are "constancy, strength, dose response and biological plausibility, leaving behind coherence, specificity, analogy and (interestingly) temporality" [131]. Moreover, this view gives substance to Susser's claim about the intimate connection between the use of causal criteria and the development of a "grammar for a pragmatic epidemiology" [154]. At the same time, this does not entail that the "inference" in IBE is nothing but a psychological inference. Acknowledging that IBE occurs within the context of inquiries about cause-and-effect relations whose goals and practices are broadly delimited by psychological, sociological and historical characteristics is not the same as saying that the inferences have no logical character. IBE still falls on the logical side of the logical/psychological dichotomy of inferences discussed earlier in the context of deductive and inductive inferences.

Of course, this still leaves a methodological issue unresolved and in need of further investigation. Even with a particular kind or sense of cause set as part of the background framework for our inquiry, how do we "know" whether applications of a set of criteria to the available (and relevant) data (such as the Bradford Hill criteria) really result in the best explanation? After all, if we had started out with different criteria, then it is possible that the explanation on which we settled would be a different one. Peirce's answer to this question in the case of abduction was that the end/goal of abduction is, "through subjection to the test of experiment, to lead to the avoidance of all surprise" and to the establishment of a productive way of interacting with the world [160]. We can tell an analogous story about the use of Bradford Hill criteria in IBE. What supports the use of the Bradford Hill criteria (or some weighted subset of the criteria) in IBE two-fold. First, the hypotheses discovered by satisfactions of the criteria in IBE are testable (by use of deductive and inductive inferences, where the concept of "test" is appropriate). If the hypotheses were not testable, this would give good reasons for selecting another set of criteria or differently weighting the criteria we had been using. Second, if true, the hypotheses discovered by satisfactions of the criteria in IBE successfully resolve outstanding problems we have that were the source of our inquiries into causes. Thus, the "justification", if one wants to use that word, of using Bradford Hill criteria in IBE is fallibilist and pragmatic. It is not likely that this will satisfy people who want some formal justification for using the criteria, but this kind of pragmatic justification seems entirely appropriate and sensitive to the different purposes that motivate our inquires into causes. After all, within the IBE framework, various weightings of the Bradford Hill criteria function as "causal values", in Poole's nicely captured sense, reflecting differing (though more or less shared) interests in making causal claims, differing (though more or less shared) concepts of cause, and differing (though more or less shared) standards of what counts as a causal measure [161].

## Conclusion

Research in epidemiology and the health sciences continues to make use of criteria such as the Bradford Hill "aspects of association" in making causal inferences based on observational data. The idea of much of this research is that using satisfactions of Bradford Hill criteria justifies the causal claims that are the conclusions of such inferences. This research ranges from clinical research in pediatric nephrology [162], to the relationship between "the parenchymal pattern of the breast seen on mammographic examination and risk of breast cancer" [163], to pharmacovigilance [164]. However, as argued above, such research is ill served by the use of the Bradford Hill criteria when the inferences in which they are used are either deductive or inductive causal inferences. If correct, then what options are available for researchers wanting to make justified causal claims? One possibility is to accept a variation of Russell's 1912 claim in his presidential address to the Aristotelian Society and say that the word 'cause' is so "inextricably bound up with misleading associations" as to make its complete extrusion from the scientific vocabulary desirable [165]. A second possibility is to say that if we want truly causal claims, then we should restrict our attention to data from properly conducted randomized controlled experimental studies. However, each of these two conclusions is, in its own way, too Draconian.

Regarding the first possibility, following Cartwright, it seems that we need causal concepts to distinguish between effective and non-effective strategies [166]. To use an example by Field, although there is a high statistical correlation between smoking and lung cancer, taking an anti-cancer drug is not an effective strategy for quitting smoking, which suggests that concept of cause plays a crucial role in distinguishing effective from ineffective strategies [167]. Thus, the cost of expunging "causal talk" from the sciences would be to undermine the practical goals of science, as well as the hope of using the results of scientific inquiry to create beneficial policies and help in making sound legal decisions. Regarding the second possibility, not only would this restrict causal claims to a very narrow range of data (excluding, for example, studies that use survey data), it also assumes that properly conducted RCTs really do justify causal claims. However, as discussed previously, this assumption is subject to a variety of practical and methodological difficulties [30,41,42], not the least of which is that, as Cartwright writes, the method of randomized controlled experiments may tell us something about causal relations in the very specific circumstances of the experiment, but "tells us nothing about what the cause does elsewhere" [107].

Rather than accepting either of the possible Draconian conclusions, I have argued in this paper that there is an alternative account of the role of the Bradford Hill criteria (and of causal criteria more generally). The problems associated with the use of causal criteria are due to supposing that their satisfactions play a role in either deductive or inductive causal inferences. Given the long tradition of dichotomizing logical inferences into deductive and inductive inferences, and supposing that the dichotomy is an exhaustive one, this is a natural supposition. However, by acknowledging and understanding a kind of logical inference, crucial in the "logic of discovery", that is neither deductive nor inductive, and by placing applications of the Bradford Hill criteria in this framework, the framework of inference to the best explanation, we find a new and important role for the criteria. Applications of the criteria, with a recognition that the criteria may change in content or in the emphasis placed on individual criteria depending on the conception of cause which motivates the inquiry about causal relations, play a crucial role in the discovery and justification of what hypothetical causal claims merit further, detailed study. What kind of further study is that? Part of the value of the role of causal criteria presented in this paper is that this question remains an open one, and that the use of causal criteria complements many possible approaches that one may take to the task of justifying the claim that it is true (or false) that a statistical association is a causal relation. Satisfactions of the Bradford Hill criteria, in the IBE framework described in this paper, do **not **permit inferring that a statistical association is a causal relation. Instead, such satisfactions only justify claiming that, **if **true, the hypothetical identification of a statistical association as a causal relation is the best explanation supported by the data [136,168]. Thus, satisfactions of the Bradford Hill criteria in the IBE framework provide a propaedeutic to further, statistical analyses of causal claims. As an example, for those interested in using Bayesian methods [169,170], the information provided by satisfactions of the Bradford Hill criteria in an IBE framework may contribute to the specification of the needed prior probabilities [136,142,171]. Once applications of causal criteria in an IBE framework present us with causal hypotheses that merit further study, only careful and reflective analyses using the appropriate methodological safeguards and statistical tools will lead to justified claims about the truth or falsity of those hypotheses.

## Competing interests

The author declares that they have no competing interests.
